# Insulin Reduces Inflammation by Regulating the Activation of the NLRP3 Inflammasome

**DOI:** 10.3389/fimmu.2020.587229

**Published:** 2021-02-19

**Authors:** Yu-Wei Chang, Ling-Chien Hung, Yu-Cheng Chen, Wen-Hung Wang, Chun-Yu Lin, Hsin-Han Tzeng, Jau-Ling Suen, Yen-Hsu Chen

**Affiliations:** ^1^ College of Medicine, Graduate Institute of Medicine, Kaohsiung Medical University, Kaohsiung, Taiwan; ^2^ Department of Laboratory, Taitung Hospital, Ministry of Health and Welfare, Taitung, Taiwan; ^3^ Division of Infectious Disease, Department of Internal Medicine, Kaohsiung Medical University Hospital, Kaohsiung Medical University, Kaohsiung, Taiwan; ^4^ School of Medicine, Graduate Institute of Medicine, Sepsis Research Center, Research Center of Tropical Medicine and Infectious Disease, Kaohsiung Medical University, Kaohsiung, Taiwan; ^5^ Research Center of Environmental Medicine, Kaohsiung Medical University, Kaohsiung, Taiwan; ^6^ Department of Medical Research, Kaohsiung Medical University Hospital, Kaohsiung, Taiwan; ^7^ Department of Biological Science and Technology, College of Biological Science and Technology, National Chiao Tung University, Hsinchu, Taiwan; ^8^ Institute of Medical Science and Technology, National Sun-Yet University, Kaohsiung, Taiwan

**Keywords:** insulin, nucleotide binding oligomerization domain (NOD)-, LRR-, and pyrin domain-containing protein 3 (NLRP3), inflammasome, ASC, immunomodulation

## Abstract

The NOD-, LRR-, and pyrin domain-containing protein 3 (NLRP3) inflammasome is the platform for IL-1β maturation, aimed at mediating a rapid immune response against danger signals which must be tightly regulated. Insulin is well known as the critical hormone in the maintenance of glucose in physiologic response. Previous studies have proved insulin has the anti-inflammatory effect but the molecular mechanism of immunomodulation provided by insulin is not clear so far. Here we investigated whether insulin reduces inflammation by regulating the NLRP3 inflammasome. In the present study, we used LPS and ATP to induce the intracellular formation of the NLRP3 inflammasome. Insulin inhibited the secretion of IL-1β by preventing the assembly of the ASC in THP-1 cells and human CD14^+^ monocyte-derived macrophages. The phosphorylation status of Syk, p38 mitogen−activated protein kinase (MAPK) and ASC were altered by insulin. These effects were attenuated in THP-1 cells transfected with small interfering RNA targeting insulin receptors. *In vivo*, administration of glucose–insulin–potassium reduced serum IL-1β level, intestinal ASC speck formation, local macrophage infiltration and alleviated intestinal injury in mice exposed to LPS. Insulin may play an immunomodulatory role in anti-inflammation by regulating the NLRP3 inflammasome.

## Introduction

Sepsis is a life-threatening organ dysfunction caused by a deregulated immune response to infection. It is a leading cause of mortality in intensive care units and a major health problem worldwide (1). The host immune responses to sepsis are characterized by pro-inflammatory and anti-inflammatory responses. In general, pro-inflammatory responses are activated to eliminate pathogens but excessive inflammation mediates tissue damage in sepsis. On the other hand, anti-inflammatory reactions are responsible for limiting tissue injury but immune suppression results in the enhanced susceptibility to secondary infection (2). Upon early inflammatory response, monocytes and macrophages play a critical role in connecting the innate and adaptive immunity during sepsis. They also participate in releasing pro-inflammatory cytokines; however, dysregulated function of these important cell subsets contributes to a “cytokine storm” and causes organ dysfunction (3). Until recently, several randomized clinical trials investigating agents for the treatment of sepsis, such as anti-TNF and anti-LPS, have failed (4). Therefore, the development of new potential therapeutic strategies against sepsis is of crucial importance.

Inflammasomes, receptors and sensors of the innate immune system, play an important role in mediating a rapid immune response against infections caused by pathogens and tissue damage (5). The assembly of the complex is initiated by the activation of pattern recognition receptors (PRRs), including the AIM2-like receptors and the nucleotide binding oligomerization domain (NOD)-like receptors (NLRs). Among the numerous PRRs identified, NLRP3 is the most extensively characterized (5, 6). The NLRP3 inflammasome is the platform for caspase-1 activation and IL-1β maturation. It consists of the NLRP3 scaffold, the ASC adaptor, and pro-caspase-1 (7). The multiprotein complex is activated upon exposure to the board spectrum of stimuli, including fungi (8), bacteria (9, 10), virus (11, 12), crystal particles (13), and environmental particles (14). Although activation of NLRP3 is critical for the clearance of extracellular pathogens and induction of the innate immune response, aberrant NLRP3 activity participates in the development of inflammation-related diseases, such as type II diabetes, gout, Alzheimer’s disease, Crohn’s disease, atherosclerosis, and sepsis (15). Therefore, accumulating studies have clarified the mechanism through which the NLRP3 inflammasome is regulated (16, 17); however, the role of NLRP3 inflammasome in sepsis awaits further investigations.

Insulin is a critical hormone that maintains blood glucose levels by facilitating cellular glucose uptake, regulating carbohydrate, lipid and protein metabolism and promoting cell division and growth through its mitogenic effects (18). Moreover, numerous experiments have established that insulin exerts glucose homeostatic and anti-inflammatory effects. Pretreatment with insulin inhibits the activation of NF-κB and expression of pro-inflammatory cytokines to alleviate inflammatory response *in vitro* and *in vivo* (19–21). However, the molecular mechanism involved in the improvement of sepsis outcome by insulin remains unclear. In the present study, we explored the immuno-modulatory role of insulin in sepsis and demonstrated that insulin inhibited the inflammatory response by reducing the oligomerization of the ASC, leading to attenuation of inflammasome activation during sepsis.

## Materials and Methods

### Cell Culture

The THP-1 human leukemia monocytic cell line was purchased from Biosource Collection and Research Center (Taiwan) and maintained in RPMI 1640 medium (Mediatech, Manassas, VA, USA) supplemented with 10% FBS and antibiotics (100 U/ml penicillin, 100 μg/ml streptomycin, and 0.25 g/ml amphotericin B).

Human CD14^+^ monocytes were isolated from donor peripheral blood mononuclear cells (PBMCs) as previously described (22). In brief, 20 ml of each vein blood sample from volunteers was collected into sterile tubes containing EDTA. First, PBMCs were obtained by gradient centrifugation with Ficoll–Hypaque (GE Healthcare, Wauwatosa, WI, USA). Human CD14^+^ cells were subsequently isolated through the column with anti-CD14 microbeads (Miltenyi BioTec GmbH, Bergisch Gladbach, Germany). Cells were subsequently washed twice with cold PBS and resuspended in RPMI 1640 medium (Mediatech) supplemented with 10% FBS and antibiotics. For the purity measurement of CD14^+^ cells, the hematology analyzer (XS-800i™; Sysmex, Kobe, Japan) was used to evaluate the cell population of the isolated cells. CD14^+^ cells showed >90% of purity compared with control PBMCs. We further differentiated the purified human CD14^+^ monocytes into macrophages by adding recombinant human granulocyte-macrophage colony-stimulating factor (GM-CSF) (10 ng/ml) (PeproTech, Rocky Hill, NJ, USA) for 7 days at 37°C in 5% CO_2_. The human blood samples were collected anonymously following approval of the project by the Institutional Review Board of Kaohsiung Medical University Hospital (KMUH-IRB-20170045).

### Animal Model

Male C57BL/6 mice (age: 6–8 weeks) were purchased from the National Laboratory Animal Center (Taipei, Taiwan), and maintained in a pathogen-free facility. The animal experimental protocol was approved by the Institutional Animal Care and Use Committee of the Kaohsiung Medical University (IACUC permit number: 106186) and was performed based on the guidelines and regulations of the institution. The mice were randomly allocated into one of four groups (n=7 per group) as follows: PBS control group, LPS exposure group, glucose–potassium (GK) solution plus LPS group (GK/LPS), and glucose–insulin–potassium (GIK) solution plus LPS group (GIK/LPS). To evaluate the insulin effect but avoid hypoglycemia and hypokalemia *in vivo*, we used the GIK solution, which is consisted of glucose (400 mg/kg), insulin (50 μg/kg), and potassium chloride (64 μEq/kg) (23), to observe the effect of insulin *in vivo*. The GK solution, the same components as those of GIK except insulin, was used as vehicle control. Briefly, naive mice (PBS control group) were primed through i.p. injection with sterile PBS (total volume: 100 μl). The GK/LPS and GIK/LPS groups were i.p. injected with GK and GIK solution for 30 min, respectively. Finally, after LPS (10 mg/kg) i.p. injection for 6 h, the mice were sacrificed.

Subsequently, a GlucoSure Blood Glucose Monitoring System (APEXBIO, Hsinchu, Taiwan) was used to monitor the blood glucose levels. The serum samples obtained from the hearts of mice were used to determine the levels of cytokines. In addition, the sera biochemistry profiles were determined using a cobas c311 analyzer (Diagnostics Roche, Rotkreuz, Switzerland). The small and large intestine of mice were obtained and fixed in 4% formaldehyde. Organ embedding in paraffin, tissue sectioning, hematoxylin and eosin (H&E) staining, and immunohistochemistry (IHC) staining were performed by Litzung Biocompany Inc. (Kaohsiung, Taiwan). The severity of intestinal injury after the i.p. administration of LPS was quantified in histological sections of the terminal ileum by a pathologist (blinded to the study groups) who assigned a score according to the International Harmonization of Nomenclature and Diagnostic Criteria scale as previously reported (24).

### Immunoblotting Analysis

After pretreatment with insulin or vehicle (HEPES) for 30 min, THP-1 cells were exposed to LPS (1 μg/ml) for 24 h. Prior to cell lysate harvest, the cells were additionally stimulated with ATP (5 mM) for 30 min. Subsequently, the cells were washed in PBS and resuspended in cell lysis buffer supplemented with a protease inhibitor cocktail. After centrifugation at 15,000 rpm for 30 min, the protein lysate was harvested and the total protein concentration was determined. An equal amount (30 μg) of protein was resolved through 10%–12% SDS-PAGE electrophoresis. Subsequently, the proteins were transferred onto a PVDF membrane. After blocking in tris-buffered saline with Tween 20 buffer containing 2% bovine serum albumin, the membrane was probed with specific antibodies as listed in **Table 1**.

**Table 1:** The sources of the antibodies used in the study.

Antibodies	Source	Identifier
Anti-NLRP3	Cell Signaling	Cat# 13158
Anti-caspase-1	Cell Signaling	Cat# 2225
Anti-cleaved caspase-1	Cell Signaling	Cat# 4199
Anti-IL-1β	Cell Signaling	Cat# 12703
Anti-cleaved IL-1β	Cell Signaling	Cat# 83186
Anti-ASC	MEDICAL & BIOLOGICAL LABORATORIES	Cat# D086-3
Anti-phospho-ASC	ECM Biosciences	Cat# AP5631
Anti-phospho-p38	Cell Signaling	Cat# 9211
Anti-phospho-JNK	Cell Signaling	Cat# 9251
Anti-phospho-ERK	Cell Signaling	Cat# 9101
Anti-phospho-Syk	ORIGENE	Cat# TA325923
Anti-insulin receptor	Cell Signaling	Cat# 3025
Anti-IGF1 receptor	Cell Signaling	Cat# 9750
Anti-actin	Millipore	Cat# MAB1501

### ASC Oligomerization Assay

The formation of ASC specks in the cell lysates was determined as previously described (25, 26). For the harvest of lysates, THP-1 cells were lysed in Buffer A (20 mM HEPES-KOH, pH 7.5, 10 mM KCl, 1.5 mM MgCl_2_, 1 mM EDTA, 1 mM EGTA, 320 mM sucrose) supplemented with a protease inhibitor cocktail. After cell lysis, a 21-gage needle was used to disrupt the cells in the total lysates (30 repetitions). The lysates were subsequently centrifuged at 300×g for 8 min. The supernatants were transferred to sterile empty Eppendorf tubes without disturbing the nuclear pellets and diluted with 1 volume of CHAPS buffer (20 mM HEPES-KOH, pH 7.5, 5 mM MgCl_2_, 0.5 mM EGTA, 0.1% CHAPS) supplemented with a protease inhibitor cocktail. After centrifugation at 2,400×g for 8 min at 4°C, the supernatants were removed. The pellets were washed twice with 0.5 ml ice-cold PBS and resuspended in 30 μl CHAPS buffer. DSS crosslinker material was added at a final concentration of 2 mM and incubated at 37°C for 30 min. Subsequently, the protein lysates were resolved through 12% SDS-PAGE electrophoresis, and the levels of ASC were determined *via* western blotting.

### Immunofluorescence Staining

Cells were washed, fixed, and incubated with anti-ASC antibodies overnight at 4°C, and finally incubated with an Alexa Fluor 488-conjugated secondary antibody. Cell nuclei were specifically stained with DAPI. Finally, a Zeiss LSM510 META laser scanning microscope (Carl Zeiss, Germany) was used to visualize the results under the oil immersion objective.

### Lactate Dehydrogenase Release Assay

We used the LDH quantitative assessment assay to evaluate the level of LDH release and determine the cell death (27). Based on the instructions provided by the manufacturer, the cell supernatants were centrifuged at 500×g for 5 min at 4°C and the dead cells were subsequently removed. The LDH cytotoxicity assay kit (Pierce Biotechnology, USA) was used and the absorbance was read at 490 nm. The results were calculated and presented as the percentage of LDH release and cell death.

### Enzyme-Linked Immunosorbent Assay

THP-1 cells and isolated human CD14^+^ monocytes were plated at a density of 4×10^5^ cells per well in 12-well plates. After pretreatment with different concentrations of insulin for 30 min, the cells were treated with LPS (1 μg/ml) for 24 h. The cells were additionally stimulated with ATP (5 mM for 30 min) or nigericin (10 μM for 1 h), followed by harvesting of the supernatants. The levels of IL-1β (Cat# 88726188), IL-6 (Cat# 88706688), and TNF-α (Cat# 88734688) were measured using ELISA kits (Thermo Fisher).

### Quantitative Real-Time Polymerase Chain Reaction

The RNA was extracted from THP-1 cells using the RNAspin Mini RNA Isolation Kit and reverse-transcribed to cDNA using the RT reagent kit. Quantitative real-time PCR analysis was performed using a LightCycler (Roche Diagnostics, Mannheim, Germany) to evaluate the levels of target mRNA. We used the ΔΔCt method to calculate the expression of target genes. All data of the quantitative PCR were normalized to the levels of GAPDH, the housekeeping gene for THP-1 cells (28). The primer sequences used in the quantitative PCR analysis were listed in **Table 2**.

**Table 2:** Primer sequences for RT-qPCR.

Primer	Sequence
caspase-1	Forward: 5’-CTTCCTTTCCAGCTCCTCAGGCA-3’Reverse: 5’-CGTGTGCGGCTTGACTTGTCC-3’
IL-1β	Forward: 5’- AGGCACAAGGCACAACAGGCTG-3’Reverse: 5’- GTCCTGGAAGGAGCACTTCATCTGT-3’
INSR	Forward: 5’-AACCAGAGTGAGTATGAGGAT-3’Reverse: 5’-CCGTTCCAGAGCGAAGTGCTT-3’
IGF1R	Forward: 5’-TCAGCGCTGCTGATGTGT-3’Reverse: 5’-GGCTCATGGTGATCTTCTCC-3’
GAPDH	Forward: 5’-TCCACCACCCTGTTGCTGTA-3’Reverse: 5’-ACCACAGTCCATGCCATCAC-3’

### RNA Interference

The target siRNAs were purchased from Thermo Fisher Scientific and transfected into THP-1 cells using the Lipofectamine RNAiMAX reagent as previously described (29). For efficient knockdown, the cells were incubated for 48 h before treatment.

### Statistical Analysis

All quantification data from the present study are represented as mean ± standard deviation from experiments performed at least 3 independent times under identical conditions. Statistical analysis was performed by GraphPad Prism 7.0 software. Two tailed *t*-tests was used for two groups and ANOVA with Dunnett’s multiple comparisons test was used for more than two groups. A *p*-value <0.05 indicated a statistically significant difference.

## Results

### Insulin Suppresses the Activation of the Nucleotide Binding Oligomerization Domain-, LRR-, and Pyrin Domain-Containing Protein 3 Inflammasome

**Figure 1:**
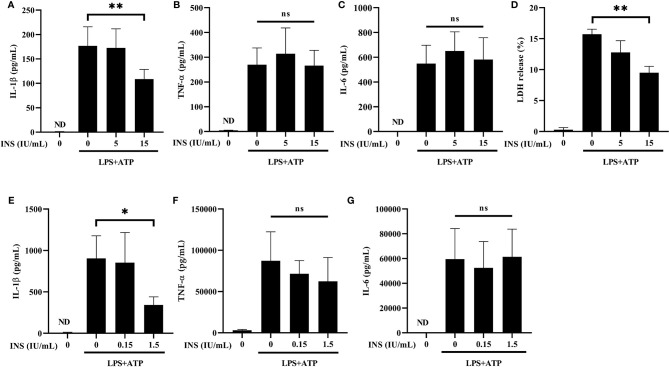
Insulin inhibits the secretion of NLRP3-mediated IL-1β, but not that of TLR-mediated TNF-α and IL-6. **(A–C)** Determination of IL-1β **(A)**, TNF-α **(B)** and IL-6 **(C)** levels in the supernatants obtained from LPS (1 μg/ml for 24 h) plus ATP (5 mM for 30 min)-stimulated THP-1 cells pretreated with insulin in a dose-dependent manner (0, 5, and 15 IU/ml) for 30 min. **(D)** Pyroptosis of THP-1 cells stimulated as in **(A)** was evaluated by LDH release assay. The present result of LDH release (%) depicted the calculated cytotoxicity. **(E-G)** ELISA assay was used to determine the production of IL-1β **(E)**, TNF-α **(F)**, and IL-6 **(G)** in the CD14^+^ isolated primary macrophages. CD14^+^ cells in the treatment group were pretreated with insulin in a dose-dependent manner (0, 0.15, and 1.5 IU/ml) for 30 min and subsequently stimulated by LPS (1 μg/ml) and ATP (5 mM). The quantification data are presented as mean values (± SDs) derived from experiments performed 3 independent times under identical conditions; *p < 0.05, **p < 0.01 (one way ANOVA with Dunnett’s multiple comparisons test). INS, insulin; LPS, lipopolysaccharide; ND, non-detectable; ns, non-significance.

NLRP3 activation requires LPS priming and ATP stimulation for the final assembly of the inflammasome complex, which cleaves pro-caspase 1 and releases the mature IL-1β (30). We used LPS plus ATP-stimulated THP-1 cells to establish an *in vitro* condition of inflammasome activation. Initially, we examined whether insulin pretreatment inhibits the release of pro-inflammatory cytokines *via* reducing inflammasome activation. Treatment with insulin significantly inhibited the expression of IL-1β in the THP-1 cells stimulated with LPS and ATP (**Figure 1A**). The consistent result was also observed that insulin reduced the IL-1β production of the THP-1 cell treated by LPS and nigericin, another NLRP3 activator (**Supplemental Figure 1A**). As a control, the production of TNF-α and IL-6 remained unchanged in insulin treatment conditions, indicating that the anti-inflammatory effect exerted by insulin is specifically associated with inflammasome activation. (**Figures 1B, C**). Subsequently, we evaluated the caspase-1-dependent programmed cell death (pyroptosis) through the LDH release assay. Following stimulation with LPS plus ATP, THP-1 cells treated with insulin exhibited a lower rate of pyroptosis than control cells (**Figure 1D**). We further used primary human macrophage to elucidate the insulin effect. CD14^+^ monocytes from human PBMCs were isolated and differentiated into macrophages through incubation with recombinant human GM-CSF. As shown in **Figure 1E**, compared with the vehicle group, a sharp reduction in IL-1β secretion was observed in the human macrophages treated with a lower concentration of insulin than those treated in THP-1 cells. This finding indicated that insulin provides more obviously effects to reduce the pro-inflammatory response in primary human cells. Consistently, insulin did not alter the expression of TNF-α and IL-6 under the same conditions (**Figures 1F, G**).These results suggest that insulin may effectively inhibit the production of pro-inflammatory cytokines and prevent the programmed cell death and this insulin effect may be associated with NLRP3 inflammasome activation.

### Insulin Inhibits the Formation of ASC Specks

**Figure 2:**
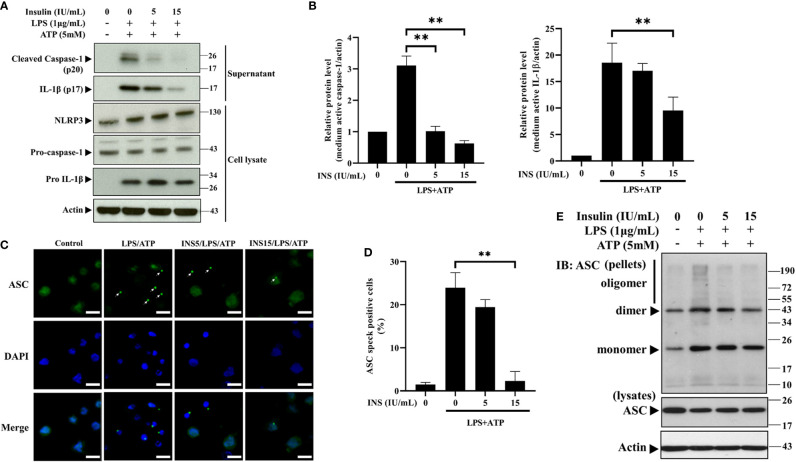
Insulin significantly reduces the NLRP3-mediated formation of the ASC pyroptosome. **(A)** Immunoblotting for caspase-1 (p20 and caspase-1 precursor), IL-1β (p17 and IL-1β precursor), and NLRP3 of the supernatant (upper panels) and cell lysates (lower panels) from THP-1 monocytes pretreated with insulin (0, 1, 5, and 15 IU/ml), followed by stimulation with LPS (1 μg/ml) for 6 h and ATP (5 mM) for 30 min. **(B)** Quantification of immunoblotting was determined by densitometric analysis, and each result was normalized to the levels of the vehicle control group. **(C)** Immunofluorescence microscopy of LPS (1 μg/ml for 24 h) and ATP (5 mM for 30 min)-stimulated THP-1 monocytes pretreated with insulin (0, 5, and 15 IU/ml) for 30 min, and subsequently immunostained for ASC (green) and DNA (DAPI, blue). Scale bars, 10 μm. White arrows indicate ASC specks. **(D)** The quantification represents the percentages of cells with an ASC speck, with ≥100 cells counted from 10 random fields in each experiment. **(E)** ASC oligomerization and redistribution assay in THP-1 cells treated as in **(A)**. Immunoblotting analysis of ASC in cross-linked pellets (upper panels) and in cell lysates (lower panels). The quantification data are presented as mean values (± SDs) derived from experiments performed three independent times under identical conditions; Immunoblotting results as shown in **(A, E)** are one representative of three independent experiments; **p < 0.01 (one way ANOVA with Dunnett’s multiple comparisons test).

NLRP3 recruits pro-caspase-1 through ASC specks, leading to activation of caspase-1 and cleavage of pro-IL-1β to mature IL-1β (7). We subsequently analyzed the expression of the main components of the NLRP3 inflammasome through western blotting analysis to determine the mechanism of insulin-dependent immunomodulation during exposure to DAMPs. As expected, insulin pretreatment reduced the expression of the cleaved caspase-1 and mature IL-1β in the supernatant obtained from THP-1 cells stimulated by LPS plus ATP. In cell lysates, there were no changes in the endogenous level of the caspase-1 and IL-1β precursors (**Figures 2A, B**). The consistent data can be seen in the **Supplementary Figure 2**, mRNA levels of caspase-1 and IL-1β were not altered by insulin. The formation of ASC specks serves as a signal amplification mechanism for the inflammasome-mediated production of cytokines (31). Hence, we examined the ability of immuno-speck formation in inflammasome-associated proteins using immunofluorescence staining and the ASC oligomerization assay. **Figures 2C, D** showed the immunofluorescence images and quantitative evaluations. We found a sharp ASC speck signal in the cytoplasm following stimulation with LPS and ATP (condensed green points in **Figure 2C**). Interestingly, ASC aggregation was significantly inhibited after pretreatment with insulin (15 IU/ml). Also, ASC speck formation derived from LPS plus nigericin was inhibited by insulin (**Supplemental Figures 1B, C**). In support of the finding, the ASC oligomerization assay showed that insulin obviously inhibited the formation of ASC specks in a dose-dependent manner (**Figure 2E**). Our results suggest that insulin may prevent the formation of ASC specks without affecting the expression of the NLRP3 in inflammasome activation condition.

### Insulin Regulates the Molecular Signaling Pathway of the Nucleotide Binding Oligomerization Domain-, LRR-, and Pyrin Domain-Containing Protein 3 Inflammasome

**Figure 3:**
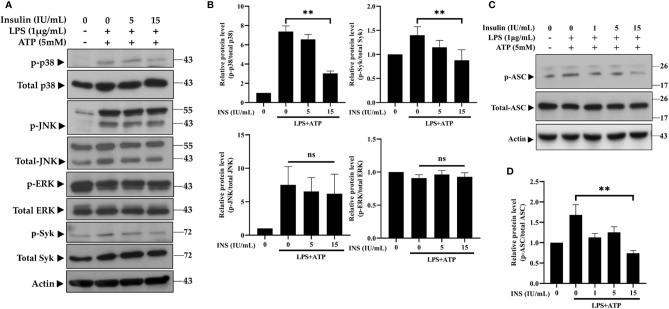
Insulin treatment regulates the molecular signaling in LPS plus ATP-stimulated THP-1 cells. **(A)** Immunoblotting analysis of MAPK and Syk kinase in the protein lysates from THP-1 cells pretreated with insulin (0, 5, and 15 IU/ml), followed by stimulation with LPS (1 μg/ml) for 2 h and ATP (5 mM) for 30 min. **(B)** Quantification of MAPK and Syk kinase was determined by densitometric analysis, and each result was normalized to the levels of the corresponding vehicle control group. **(C)** Immunoblotting analysis of phosphoryl ASC (p-ASC) in the protein lysates from THP-1 cells pretreated as in **(A)**. **(D)** Quantification of p-ASC was determined by densitometric analysis, and normalized the levels of the vehicle control. The quantification data are presented as mean values (± SDs) derived from experiments performed three independent times under identical conditions; Immunoblotting results as shown in **(A, C)** are one representative of three independent experiments; **p < 0.01 (one way ANOVA with Dunnett’s multiple comparisons test). ns, non-significance.

Syk kinase signaling in macrophages may play a critical role in immunomodulation through activation of the inflammasome (32, 33). Furthermore, both the Syk and MAPK pathways are involved in the phosphorylation and oligomerization of ASC (34, 35). Thus, we investigated the role of Syk and MAPK in the anti-inflammatory response induced by insulin. As can been seen in **Figures 3A, B**, the phosphoryl activities of Syk were significantly reduced after pretreatment with insulin in LPS plus ATP-stimulated THP-1 cells. While observing the MAPK activity, we found that p38 MAPK were obviously blocked following the treatment of insulin; however, there were no effects on the phosphorylation of JNK and ERK. We also confirmed the insulin significantly abrogated the phosphorylation of ASC (**Figures 3C, D**). Our results showed that insulin may inhibit ASC phosphorylation, by reducing the phosphorylation of Syk and p38 MAPK.

### Insulin Modulates the Expression of ASC-IL-1β Axis *via* the INSR and IGF1R

**Figure 4:**
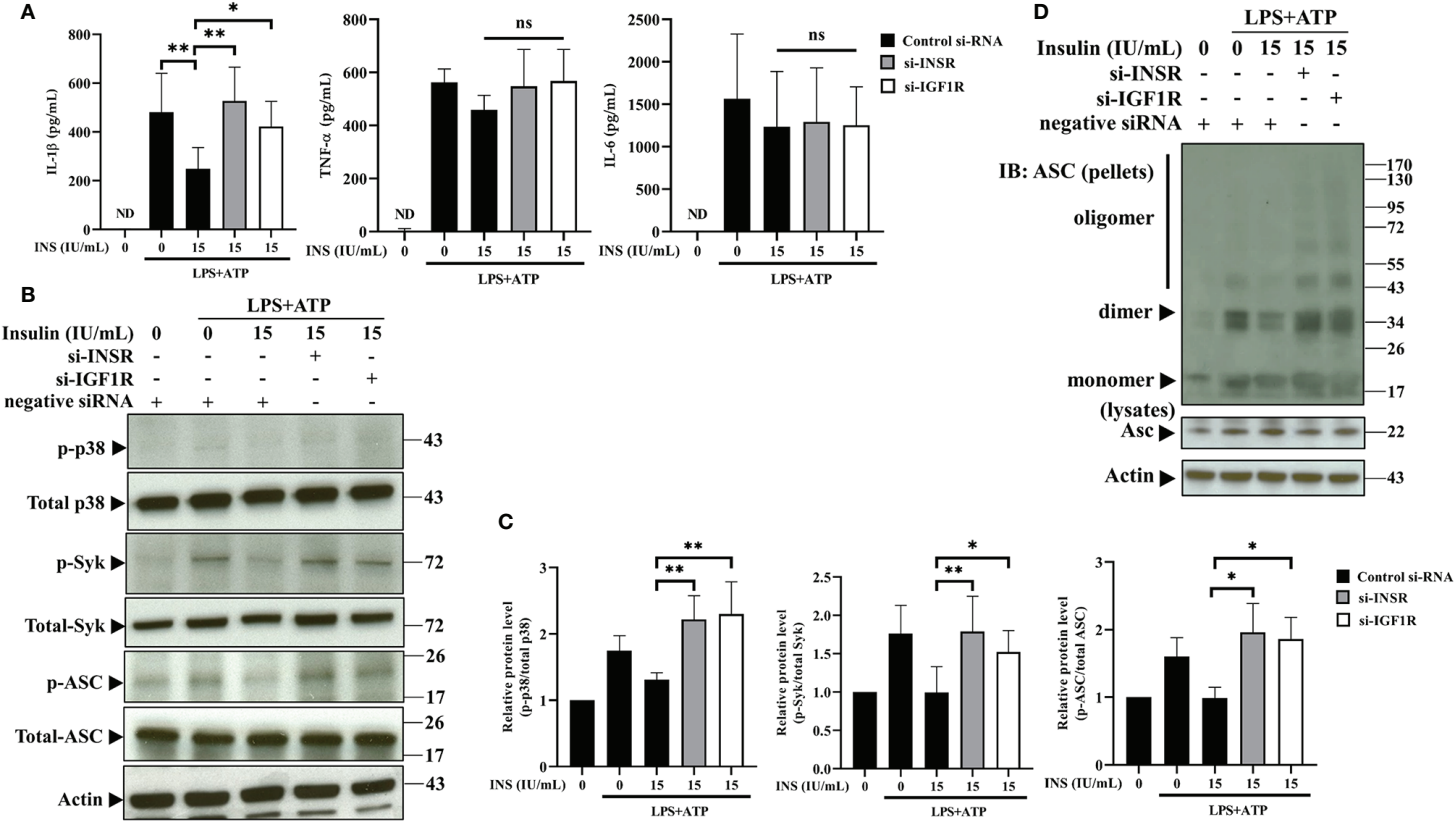
Insulin modulates the expression of ASC-IL-1β axis *via* the insulin receptor and insulin-like growth factor-1 receptor. **(A)** Cytokine ELISA assay for the levels of IL-1β, TNF-α, and IL-6 in the supernatants of THP-1 cells transfected with INSR-, IGF1R-, or negative control siRNA, followed by pretreatment with insulin (15 IU/ml for 30 min) and stimulation with LPS (1 μg/ml for 4 h) plus ATP (5 mM for 30 min). **(B)** Immunoblotting analysis of p38, Syk, and ASC in the protein lysates from THP-1 cells pretreated as in **(A)**. **(C)** Quantification of p38, Syk, and ASC were determined by densitometric analysis, and each result was normalized to the levels of the corresponding vehicle control groups. **(D)** ASC oligomerization and redistribution assay in THP-1 cells treated as in **(A)**. Immunoblotting analysis of ASC in cross-linked pellets (upper panels) and cell lysates (lower panels). The quantification data are presented as mean values (± SDs) derived from experiments performed three independent times under identical conditions (n=3); Immunoblotting result as shown in **(B, D)** are one representative of three independent experiments; *p < 0.05, **p < 0.01 (one way ANOVA with Dunnett’s multiple comparisons test). ns, non-significance.

To further confirm the immunomodulation of NLRP3 inflammasome derived from insulin, we evaluated IL-1β secretion in response to NLRP3 activation in THP-1 cells, in which INSR and IGF1R (major receptors of insulin) had been knocked down using small interfering RNA (siRNA). Firstly, siRNA-mediated gene silencing specifically and effeIctively inhibited the expression of the target mRNA and protein (**Supplemental Figures S3A–E**). Individual depletion of INSR or IGF1R significantly reversed the NLRP3 inflammasome-mediated secretion of IL-1β, respectively; however, it did not affect the expression of TNF-α and IL-6 (**Figure 4A**). Furthermore, the suppressive effects contributed by insulin could be reversed by silencing of INSR or IGF1R, such as the phosphorylation of p38, Syk, and ASC (**Figures 4B, C**), and ASC oligomerization (**Figure 4D**). Thus, these results suggest that insulin may play an important role in modulating the activation of the ASC-IL-1β axis in NLRP3 inflammasomes.

### Insulin Suppressed the Production of Pro-Inflammatory Cytokines and Alleviated Intestinal Injury *In Vivo*

**Figure 5:**
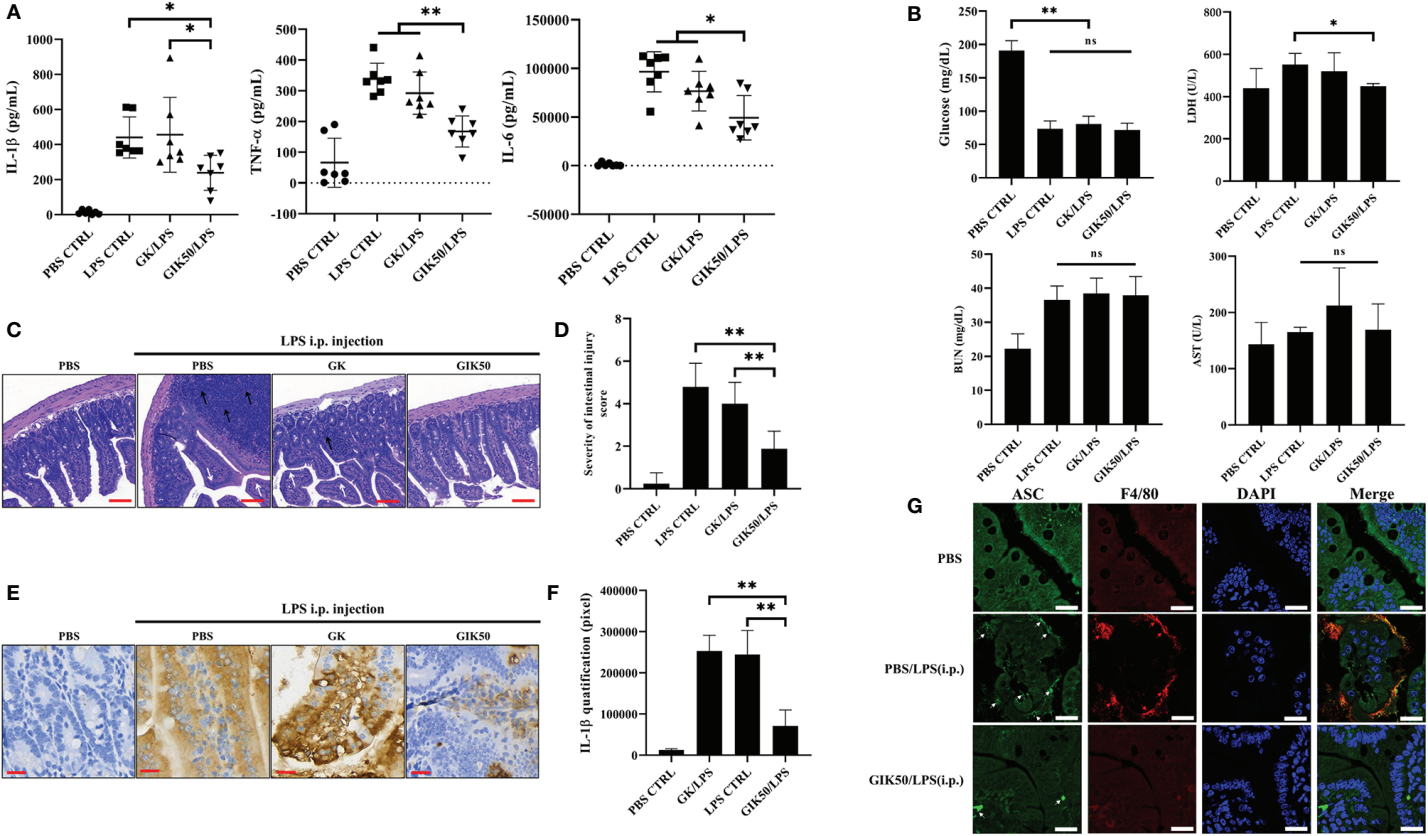
Immunomodulatory effects of insulin on systemic inflammation and intestinal injury in the LPS-treated mice. Serum samples were harvested 6 h after intraperitoneal injection with LPS, GK, or GIK50 in the first set of mice. **(A)** The serum levels of IL-1β, TNF-α, and IL-6 (n=7). **(B)** The levels of blood glucose (n=7), LDH (n=3), urea nitrogen (BUN) (n=3), and aspartate aminotransferase (AST) (n=3). **(C)** The representative images of H&E stained intestine sections depicts the infiltration of inflammatory cells (black arrows; scale bar, 100 μm) and disruption of the small intestinal mucosa (white arrows). **(D)** Quantification of the intestinal injury score was performed according to INHAND grades, as described in the section of *Materials and Methods* (n=3). **(E)** The images of immunohistochemical staining of intestine sections using the IL-1β mouse monoclonal antibody. (scale bar, 30 μm) **(F)** Quantification of IHC staining was determined in pixels (n=3). **(G)** Immunofluorescence images show ASC speck (green, white arrow) and F4/80 (red, red arrow) distribution in the small intestinal mucosa. Nuclei (blue) were revealed by DAPI. The merged images revealed the co-localization of ASC with F4/80, the cell surface markers of mice macrophages (Scale bars, 20 μm, n=3). The quantification data are represented as mean ± SDs, with “n” indicating the number of mice per group; **p < 0.01 (one way ANOVA with Dunnett’s multiple comparisons test). GK, glucose-potassium solution; GIK, glucose-insulin-potassium solution; i.p., intraperitoneal. *p < 0.05, **p < 0.01; ns, non-significance.

We elucidated the *in vivo* immune modulatory effects of insulin using a GIK solution administered to a LPS induced murine endotoxemia model (36, 37). Serum analysis showed that pro-inflammatory cytokines (including IL-1β, TNF-α, and IL-6) were effectively induced 6 h after intraperitoneal (i.p.) injection of LPS in C57BL/6 mice (**Figure 5A**). Compared with the LPS group or GK/LPS group, mice treated with GIK50 (contains 50 μg insulin/kg body weight) followed by LPS injection were significantly reduced levels of the pro-inflammatory cytokines, including IL-β, TNF-α, and IL-6. The level of blood glucose was significantly reduced following the administration of LPS, whereas GK/LPS or GIK/LPS treatment did not affect the glucose level compared to LPS-treated mice. Regarding serum biochemistry, only LDH level, as an additional pyroptosis marker (38), was significantly inhibited by treatment with GIK50, but not blood urea nitrogen (BUN) and aspartate aminotransferase (AST) (**Figure 5B**). Furthermore, the architecture of the terminal ileum was preserved in the control group (PBS control without LPS exposure) (**Figure 5C**). However, mice i.p. injected with LPS showed an obvious disruption of the small intestinal mucosa, caused by the intensive infiltration of inflammatory cells into the lamina propria, edema of the submucosa, and epithelial cell loss. Interestingly, treatment with GIK50/LPS decreased the severity of LPS-mediated intestinal injury, compared to the GK/LPS group (**Figures 5C, D**). After that, we performed immunohistochemical assessment to further observe the mucosal cytokine expression involved in the small intestine. In line with the pathologic results, GIK50/LPS treatment sharply reduced the IL-1β expression compared to the LPS or GK/LPS groups (**Figures 5E, F**). It is well known that IL-1β promotes the macrophage recruitment in the context of inflammation (39). Thus, we next tested whether insulin treatment suppressed macrophage infiltration in tissue. Further evidence can be seen in the immunofluorescence images (**Figure 5G**). ASC specks were observed around the small intestinal mucosa from LPS administrated mice and co-localized with the infiltrated macrophages, whereas GIK/LPS treatment obviously blocked the ASC speck formation and reduced the macrophages infiltration. Collectively, insulin potentially exerts anti-inflammatory effects and in turn alleviates pyroptosis in mice with LPS-induced sepsis.

## Discussion

**Figure 6:**
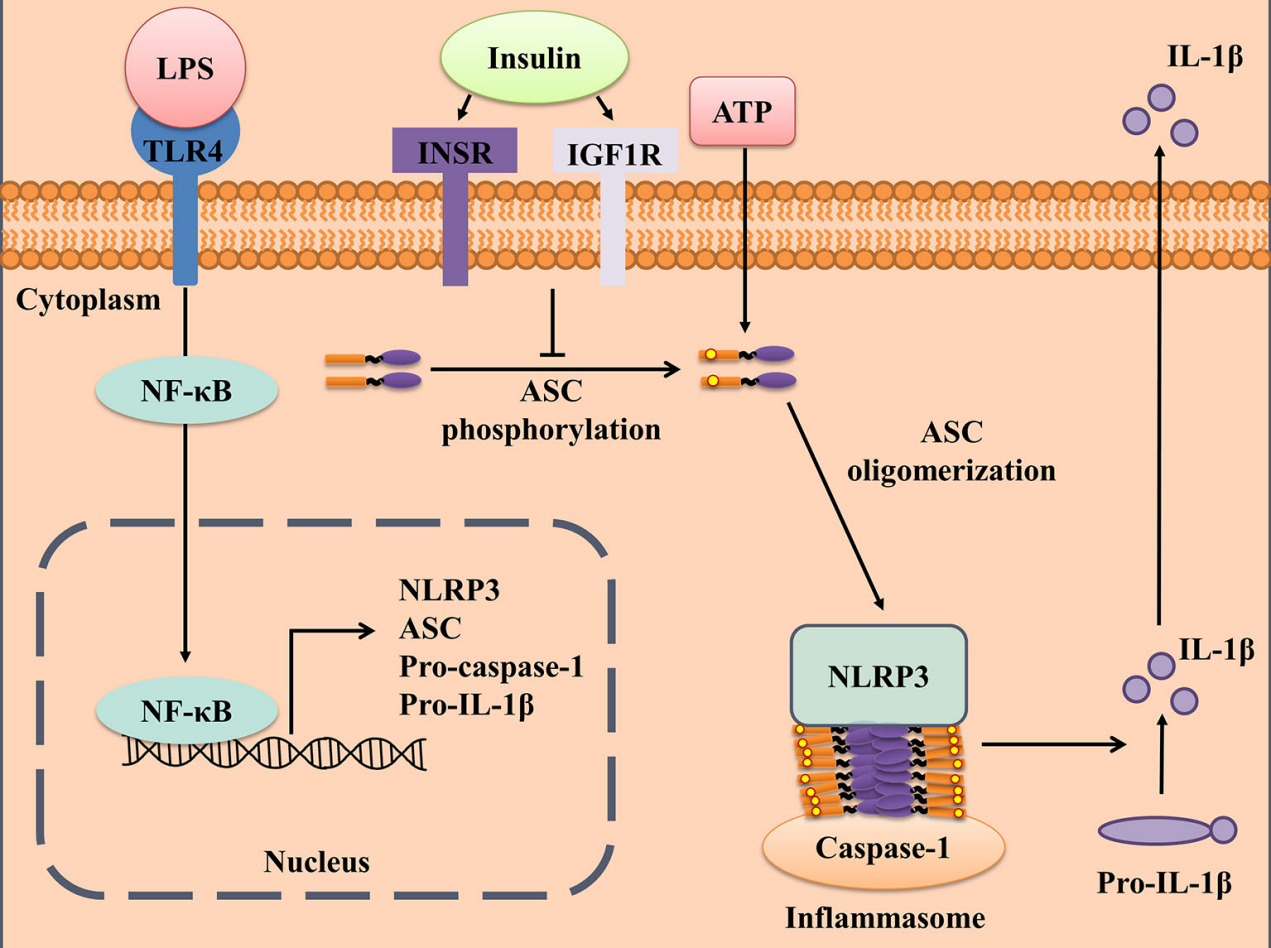
Schematic illustration of a possible mechanism for insulin reduced NLRP3 inflammasome activation. According to our results, we proposed that insulin inhibited ASC phosphorylation cascade and followed by reducing ASC oligomerization in the presence of NLRP3 stimulus. This immuno-modulatory effect derived from insulin reduced the IL-1β production and prevented pyroptosis cell death, which exerted the protective role during the danger signals exposure.

It has been shown that insulin has anti-inflammatory effects; however, the molecular mechanism remains largely unknown. The present study demonstrates that insulin exerts anti-inflammatory effect by alleviating the formation of ASC specks, to finally reduce the activation of the inflammasome and secretion of the pro-inflammatory cytokines in an INSR and IGF1R-dependent manner (**Figure 6**). To our best knowledge, this is the first study to reveal that insulin is a key regulator of NLRP3 inflammasome activation. Furthermore, the administration of insulin in mice with LPS-induced sepsis revealed that insulin effectively exerts an anti-inflammatory effect and prevented pyroptosis cell death. These findings suggest insulin may not only be the homeostasis endocrine but serve as an important role in immune-modulation in chronic metabolic or severe infectious diseases.

Insulin has been documented to exert glucose homeostatic and anti-inflammatory effects. It was capable of reducing the activation of intranuclear NF-κB activity and expression of pro-inflammatory cytokines, to alleviate inflammatory response *in vitro* and *in vivo* (19, 21, 40, 41). Furthermore, the previous studies revealed that intensive therapy with insulin improved the mortality rate in intensive care units, further indicating that treatment with insulin reduced mortality in patients with sepsis (42, 43). The evidence suggests that insulin may exert an anti-inflammatory effect by modulating the inflammatory response, and finally improves the symptoms of infection. However, studies concerning the anti-inflammatory effects of insulin have been limited in terms of the sample population or were not focused on the molecular mechanism. In the present study, we provide evidence showing the immunomodulation by insulin in the NLRP3 inflammasome pathway, which is rapidly stimulated in response to a variety of stress and infectious signals and elicits a robust inflammatory response.

Our results detail the molecular mechanism of inflammasome activation, which is regulated by treatment with insulin. The data suggest the protective role played by insulin in the inflammatory response. These findings extend the conclusion of the previous studies, suggesting that treatment with insulin therapy improves the outcome of patients with sepsis in the intensive care unit (42). In addition, the improvements noted were the results of immunomodulation contributed by insulin, which has negative feedback of inflammatory stress caused by microbes, immune disorders, and chronic diseases. Furthermore, it has been demonstrated that the effects of anti-inflammatory and organ protection gained from insulin in scalded experimental rats without controlling hyperglycemia (44). In line with the conclusion, our findings have greatly enhanced the understanding of the molecular mechanisms by which the immunomodulation derived from insulin are involved.

Binding of insulin to the α subunit of INSR induces a conformational change, followed by facilitation of the cell signal transduction, such as the binding of ATP, phosphorylation of β subunits, recruitment of intracellular substrates, and their subsequent phosphorylation (45, 46). Moreover, insulin binds to and activates the INSR, IGF1R, or INSR/IGF1R hybrid receptors with different affinities. Here, we propose that insulin modulates the activation of the NLRP3 inflammasome *via* INSR and IGF1R. Consistently, depletion of INSR or IGF1R by siRNA can reverse the inhibitory effect of insulin on the activation of the NLRP3 inflammasome (**Figure 4**). We demonstrated that INSR and IGF1R play a linked role in the dysregulation of NLRP3 inflammasome caused by insulin. Further investigations are warranted to clarify the docking conformation between insulin and the associated receptor, which can influence the development of inflammatory diseases caused by dysregulation of the NLRP3 inflammasome.

In contrast to *in vitro* effect, our results showed that *in vivo* effect of insulin on proinflammatory cytokine production is not restricted to IL-1β. The levels of TNF-α and IL-6 were also inhibited by insulin treatment in a LPS induced murine endotoxemia model. It is highly possible that the decrease of TNF-α and IL-6 was due to the significant IL-1β decrease *in vivo* as IL-1β release is a critical step in cascade induction of other pro-inflammatory cytokines (47–49). Another possible reason is that insulin may exert anti-inflammatory effect through another IL-1β-independent mechanism *in vivo*, including suppressing NF-κB pathway (21) or changing T cell polarization to Th2 (50). Thus, the levels of all three pro-inflammatory cytokines were significantly decreased in the insulin-treated model in the present study. In addition to the cascade inducing activity of IL-1β for other two proinflammatory cytokines, IL-1β can recruit macrophages in the context of inflammation (39). Therefore, the obviously less macrophage infiltration in insulin treated group is highly possible because of the decrease of IL-1β release *in vivo*.

However, this study had limitations. The results obtained in other kinds of cytosolic PRRs (e.g., NLRP1, AIM2, or NLRC4) were not representative of the entire pathophysiology encountered in sepsis. Future studies should include different DAMPs or PAMPs stimulations. This approach will comprehensively evaluate whether treatment with insulin plays a protective role in host cells receiving the inflammatory stimulators, and further establish the potential of insulin as a new therapeutic agent against sepsis.

In conclusion, insulin serves as a negative regulator of NLRP3 inflammasome activation. Our results provide compelling evidence for translational medicine to improve the clinical predicament in sepsis. Moreover, they suggest that treatment with insulin is effective against inflammatory stress. We expect the present evidence provides a new strategy for the therapeutic application of insulin and introduces an entirely new view for the comprehensive study of this field.

## Data Availability Statement

The raw data supporting the conclusions of this article will be made available by the authors, without undue reservation.

## Ethics Statement

The studies involving human participants were reviewed and approved by the Institutional Review Board of Kaohsiung Medical University Hospital (KMUH-IRB-20170045). The patients/participants provided their written informed consent to participate in this study. The animal study was reviewed and approved by the Institutional Animal Care and Use Committee of the Kaohsiung Medical University (IACUC permit number: 106186). Written informed consent was obtained from the individual(s) for the publication of any potentially identifiable images or data included in this article.

## Author Contributions

Y-WC, L-CH, and J-LS conceived and designed the experiments. Y-WC, L-CH, Y-CC, and H-HT performed the experimental works. Y-WC, W-HW, L-CH, C-YL, and J-LS analyzed and interpreted the results. Y-WC and J-LS drafted the manuscript. Y-HC is the guarantor of integrity of the entire study and responsible of editing and finally reviewing of the paper. All authors contributed to the article and approved the submitted version.

## Funding

This study is supported partially by Kaohsiung Medical University Research Center Grant (KMU-TC108B03), Ministry of Science and Technology, Taiwan (MOST 106-2314-B-037 -087 and MOST 107-2314-B-037 -079 to Y-HC), and Ministry of Health and Welfare, Taiwan (Project No. 10645 to Y-WC).

## Conflict of Interest

The authors declare that the research was conducted in the absence of any commercial or financial relationships that could be construed as a potential conflict of interest.
