# Supplementary material for: Insulin Reduces Inflammation by Regulating the Activation of the NLRP3 Inflammasome
Source: Front Immunol. 2021 Feb 19;11:587229. doi: 10.3389/fimmu.2020.587229 (PMC7933514; doi:10.3389/fimmu.2020.587229)
Supplement: Supplementary file 1 [file DataSheet_1.docx]

Supplementary Material

## Supplementary Figures


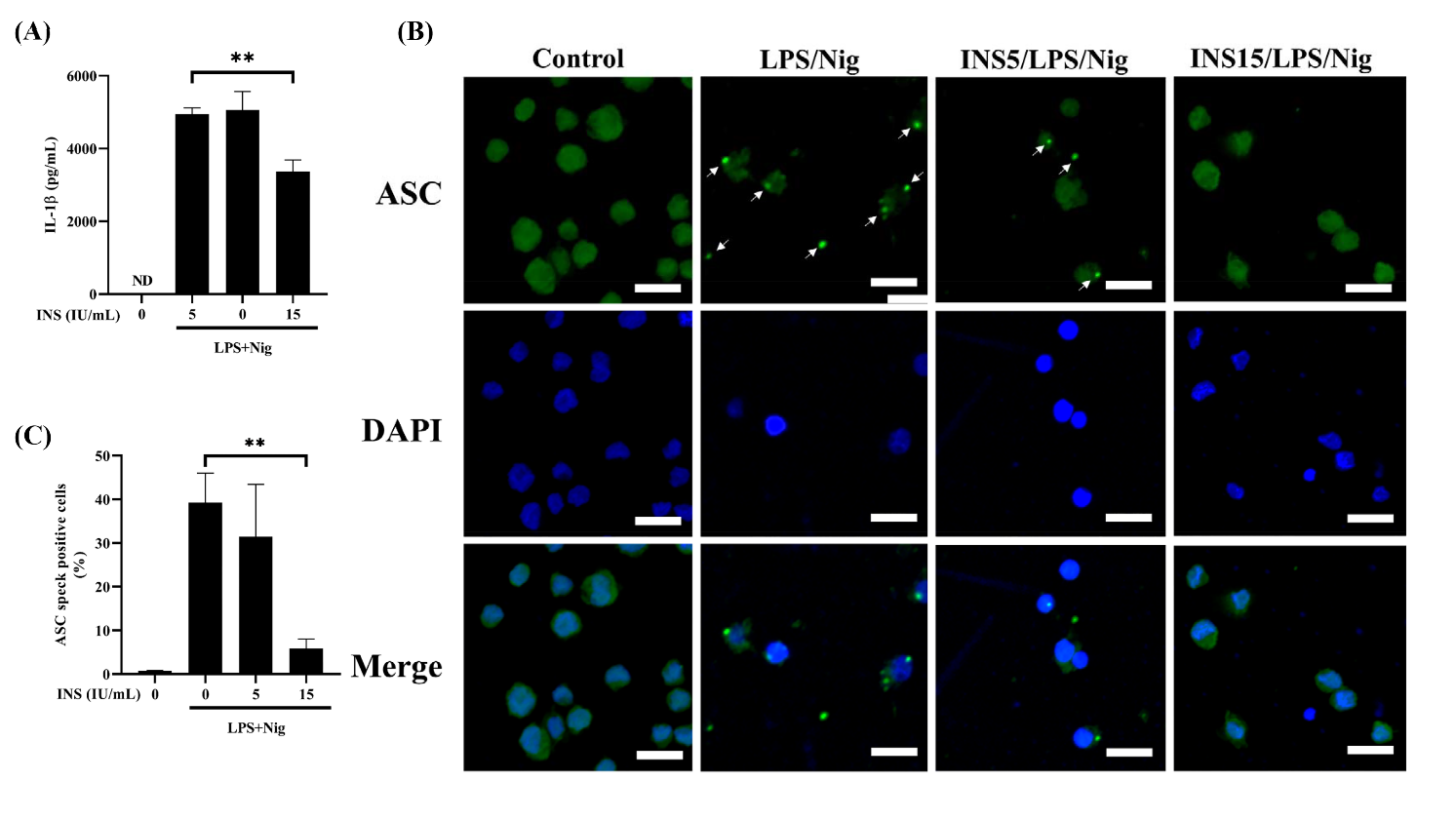


**Supplementary Figure 1. Insulin inhibited the IL-1β production and ASC specks assembling derived from LPS and nigericin stimulation.**

**(A)** Determination of IL-1β levels in the supernatants obtained from LPS (1 μg/mL for 24 h) plus nigericin (10 μM for 1 h)-stimulated THP-1 cells pretreated with insulin in a dose-dependent manner (0, 5, and 15 IU/mL) for 30 min. **(B)** Immunofluorescence microscopy in THP-1 cells treated as in (A), and subsequently immunostained for ASC (green) and DNA (DAPI, blue). Scale bars, 10 μm. White arrows indicate ASC specks. **(C)** The quantification represents the percentages of cells with an ASC speck, with ≥100 cells counted from 10 random fields in each experiment. The quantification data are presented as mean values (±SDs) derived from experiments performed 3 independent times under identical conditions (n=3); Immunoblotting results as shown in (B) are the representative of multiple experiments; **p* < 0.05, ** *p* < 0.01 (one way ANOVA with Dunnett’s multiple comparisons test). Abbreviation: INS, insulin; LPS, lipopolysaccharide; Nig: nigericin; ND, non-detectable


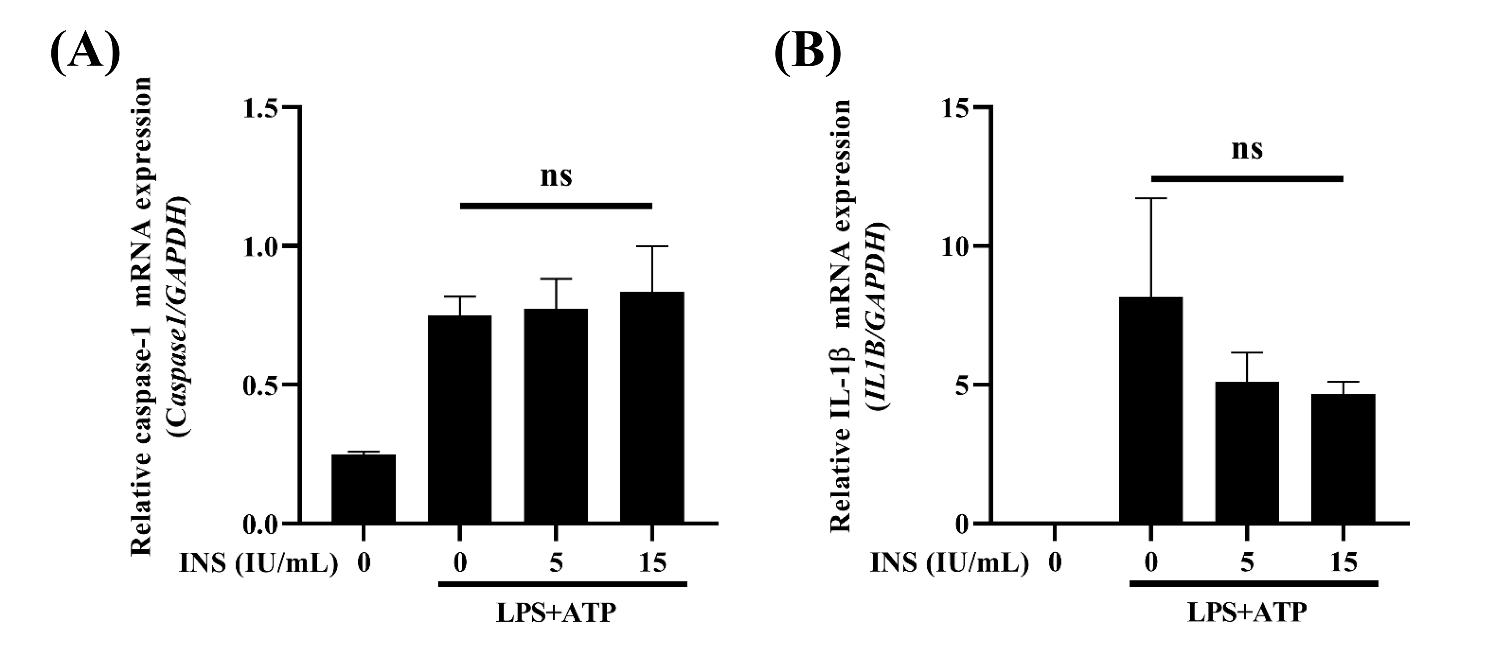


**Supplementary Figure 2.** **Insulin did not alter the mRNA expression of pro-caspase-1 and pro-IL-1β in THP-1 cells stimulated by LPS and ATP.**

**(A, B)** Quantitative PCR analysis of pro-caspase-1 (A) and pro-IL-1β (B) in THP-1 cells stimulated by LPS (1 μg/mL for 4 h) plus ATP (5 mM for 30 min)-stimulated THP-1 cells pretreated with insulin in a dose-dependent manner (0, 5, and 15 IU/mL) for 30 min. Quantification of target mRNA level was normalized with internal control levels (GAPDH). The quantification data are presented as mean values (±SDs) derived from experiments performed 3 times under identical conditions (n=3); ***p* < 0.01 (one way ANOVA with Dunnett’s multiple comparisons test). Abbreviation: ns, non-significance


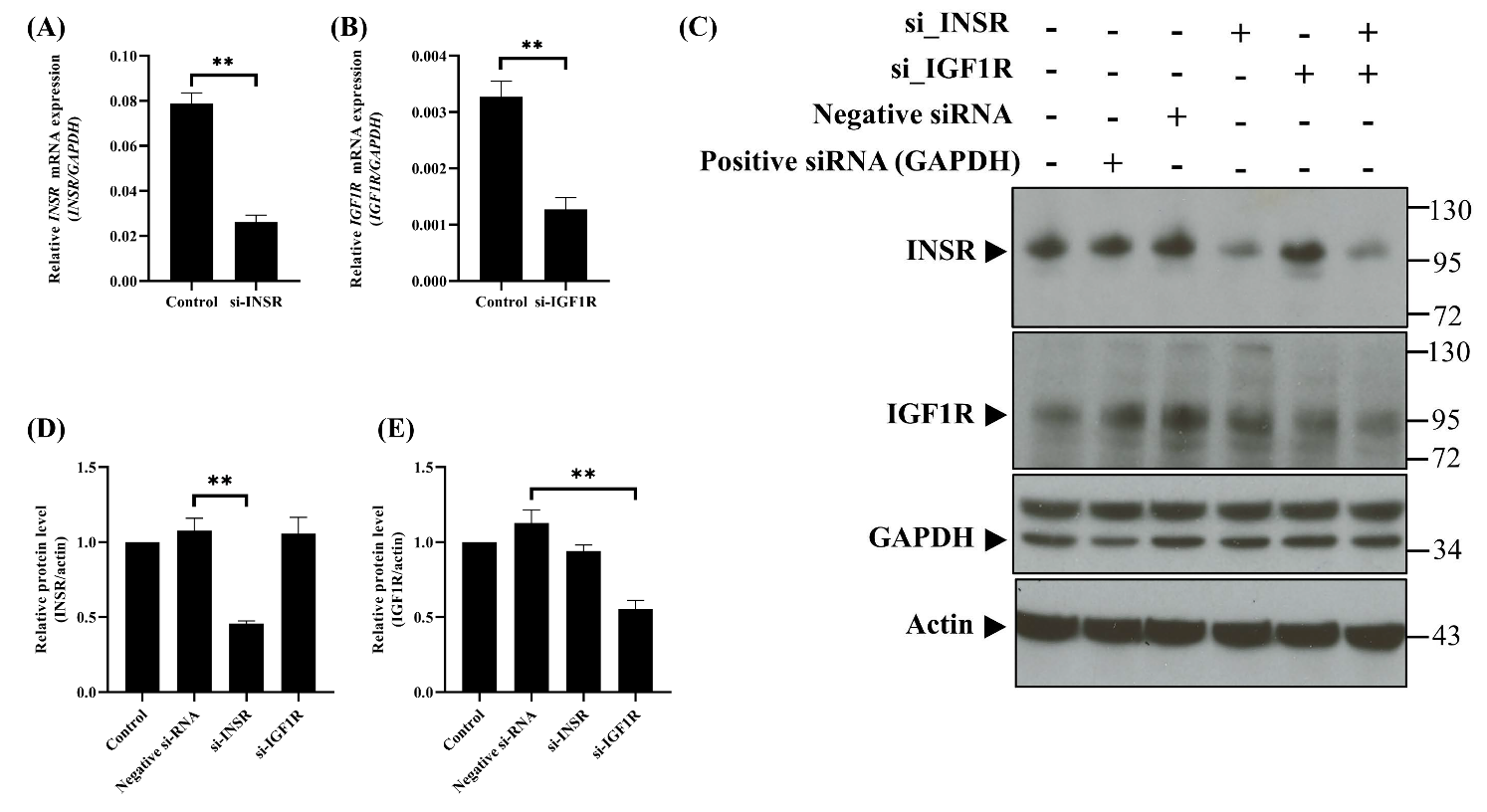


**Supplementary Figure 3.** **INSR- and IGF1R- siRNA specifically inhibited the expression of the target mRNA and protein.**

**(A-E)** Quantitative PCR (A, B) and immunoblotting analysis (C) of INSR and IGF1R in THP-1 cells transfected with INSR-, IGF1R-, negative control, or positive control siRNA. (D, E) Quantification of INSR and IGF1R was determined by densitometric analysis, and normalized with internal control levels (actin). The quantification data are presented as mean values (±SDs) derived from experiments performed 3 independent times under identical conditions (n=3); Immunoblotting results as shown in (C) is one representative of 3 independent experiments ***p* < 0.01 (two tailed *t*-test in panels A and B; one way ANOVA with Dunnett’s multiple comparisons test in panels D and E).
